# The Fate of Fat: Pre-Exposure Fat Losses during Nasogastric Tube Feeding in Preterm Newborns

**DOI:** 10.3390/nu7085279

**Published:** 2015-07-29

**Authors:** Maissa Rayyan, Nathalie Rommel, Karel Allegaert

**Affiliations:** 1 Department of Development and Regeneration, KU Leuven, Herestraat 49, 3000 Leuven, Belgium; E-Mail: maissa.rayyan@uzleuven.be; 2 Neonatal Intensive Care Unit, University Hospitals Leuven, Herestraat 49, 3000 Leuven, Belgium; 3 Translational Research Center for Gastrointestinal Diseases, KU Leuven, 3000 Leuven, Belgium; E-Mail: nathalie.rommel@med.kuleuven.be; 4 Neuro-gastroenterology and Motility, Gastroenterology, 3000 Leuven, Belgium; 5 Neurosciences, Exp ORL, KU Leuven, Herestraat 49, O & N 2, PO Box 79, 3000 Leuven, Belgium

**Keywords:** preterm, nutritional support, lipids, nasogastric tube feeding, fat delivery, absorption

## Abstract

Deficient nutritional support and subsequent postnatal growth failure are major covariates of short- and long-term outcome in preterm neonates. Despite its relevance, extrauterine growth restriction (EUGR) is still prevalent, occurring in an important portion of extremely preterm infants. Lipids provide infants with most of their energy needs, but also cover specific supplies critical to growth, development and health. The use of human milk in preterm neonates results in practices, such as milk storage, pasteurization and administration by an infusion system. All of these pre-exposure manipulations significantly affect the final extent of lipid deposition in the intestinal track available for absorption, but the impact of tube feeding is the most significant. Strategies to shift earlier to oral feeding are available, while adaptations of the infusion systems (inversion, variable flow) have only more recently been shown to be effective in “*in vitro*”, but not yet in “*in vivo*” settings. Pre-exposure-related issues for drugs and nutritional compounds show similarities. Therefore, we suggest that the available practices for “*in vitro*” drug evaluations should also be considered in feeding strategies to further reduce pre-exposure losses as a strategy to improve the nutritional status and outcome of preterm neonates.

## 1. Introduction

The nutritional management of preterm infants, especially in extremely preterm (<28 weeks) or extremely low birth weight (<1000 g) infants, should aim to result in growth patterns that approximate the patterns of intrauterine fetal growth [1]. Using this paradigm, extrauterine growth restriction (EUGR) is still prevalent, occurring in a very relevant portion of extremely preterm infants. Extrauterine growth restriction has been associated with significant caloric and protein deficits that accumulate during hospitalization, but also with slower growth velocity and major neonatal morbidities, including bronchopulmonary dysplasia, retinopathy of prematurity and subsequent impaired neurodevelopment [2]. However, it is not so obvious how to disentangle the cause and consequence. To illustrate this, we refer to a recent analysis on perinatal variables associated with extrauterine growth restriction in a longitudinal study in four neonatal intensive care units (NICU)’s in Brazil [3]. Based on perinatal data of 570 very low birth weight infants (<1500 g), maternal hypertension, male gender, growth restriction at birth, respiratory distress syndrome and length of hospital stay were significantly associated with the weight Z-score at hospital discharge. Moreover, logistic regression analysis (weight Z-score ≤−2 at hospital discharge) documented that the length of hospital stay, the presence of respiratory distress syndrome, treated patent ductus arteriosus and growth restriction at birth were retained in the final weight model [3,4]. These neonatal morbidities are commonly treated with fluid restriction, resulting in associated caloric restriction [5]. To further stress the interaction between disease and nutritional deficiency, respiratory disease in the first 28 days of life was the best predictor of reduced growth velocity in a cohort of 1187 extremely (23–27 weeks) preterm neonates in a multivariable model [6]. 

While the causes of postnatal growth restriction in preterm infants are multifactorial, it has been estimated that about 50% of the variance in early postnatal growth can be attributed to nutrition [7]. This is because the nutritional intakes provided to extremely preterm (*i.e.*, <28 weeks gestational age) or extremely low birth weight (ELBW, *i.e.*, <1000 g) neonates are commonly lower than recommended, resulting in cumulative deficits in caloric accretion, in body weight, body length and head circumference during later neonatal stay, at discharge and beyond [2]. Extrauterine growth restriction in these patients is to a certain extent similar to the neonates with intrauterine growth restriction at birth. However, and in contrast to the intrauterine variant, the extrauterine variant is a medically-induced, iatrogenic and likely avoidable complication of preterm birth.

Overall, deficient nutritional support and the subsequent postnatal growth failure are major covariates of both the short- and long-term outcomes of preterm neonates, but not always in a simple linear relationship. Rapid postnatal weight (re)gain (“catch up growth”) following nutritional restriction is associated with both positive effects on neurodevelopment outcome, as well as with the development of insulin resistance and metabolic syndrome in later life [2,5]. It is likely that minimization of early postnatal growth failure will decrease the need for catch-up growth and thereby decrease the risk of developing cardiovascular risk factors while still maintaining the positive effects on neurodevelopment, resulting in the concept of “early aggressive nutrition” [5,7].

Lipids provide infants with most of their energy needs. Consequently, dietary lipids are key for preterm neonates, and this is not limited to attaining their energy needs (9 kcal/g fat), but also covering essential supplies critical to growth, development and health. Besides quantitative aspects, qualitative aspects also matter [8,9,10]. The major portions of fat in human milk can be retrieved as triglycerides or phospholipids, with only a limited amount of cholesterol. Long-chain polyunsaturated fatty acids (LC-PUFAs) are crucial for normal development of the central nervous system and may have long-lasting effects beyond the period of dietary insufficiency. Since the formation capacity of docosahexaenoic acid (DHA) from α-linolenic acid in preterm neonates is limited and variable and because DHA is critical for normal retinal and brain development in humans, DHA should be considered to be conditionally essential and should also be present in lipid supplementation strategies in neonates, either parenteral or enteral [8,9,10].

Based on the currently available information, it seems safe to start lipid emulsions from birth onward at a rate of 2 g lipids/kg/day, although there is still the practice of using a more incremental approach [10,11]. Mixed lipid emulsions, including those containing fish oil, seem to reduce nosocomial infections in preterm infants and might reduce bile acid accumulation. Liver damage may be reduced by decreasing or removing lipids from parenteral nutrition or may be reduced by using fish oil-containing lipid emulsions containing high levels of vitamin E [10]. In early enteral studies, the amount of specific lipids in formula was chosen to produce similar concentrations of arachidonic acid and DHA as in term human milk. Recent studies report outcome data in preterm infants fed formula with DHA content 2–3-times higher than the current concentration. Overall, these studies show that providing larger amounts of DHA supplements is associated with better neurological outcome [8,9,10].

Finally, the switch from parenteral to enteral nutrition warrants specific attention. Human milk is the reference product and optimal for term neonates, but not for preterm neonates [12,13]. Fortification, either standardized or individualized/targeted, is needed to cover the needs in preterm neonates [14,15,16]. The concept, including feasibility and the day-to-day variation in macronutrients (proteins 20%, carbohydrates 13% and lipids 17%), of repeated human milk analysis and subsequent target fortification has recently been discussed in this journal [17].

Unfortunately, there seems to be a very significant and clinically-relevant difference between the lipid content in fresh human milk and the final amount of lipids administered to the intestinal track of preterm neonates.

This commentary aims to focus on these pre-exposure fat losses to further stimulate research in this specific field. We hereby aim to make the link with population-specific drug formulations and administration practices.

## 2. Development of Oral Feeding Skills

Preterm infants are still immature, including their neurologic, gastro-intestinal and respiratory system. As a part of this, the coordination of suck-swallow-respiration is not yet mature. As a result, they have difficulties establishing oral feeding. Until they achieve adequate feeding skills, tube feeding is used. Failure to establish oral feeding can lead to poor nutritional status and growth failure [18,19]. Moreover, neurodevelopmental and growth outcome in the long term depend on oral feeding competence [20]. Although there is a general consensus on the need to make the transition to oral feeding as soon as possible, a consensus on how to proceed is lacking. Feeding practices depend on local practices and beliefs [21,22]. Human milk is recommended for enteral nutrition of infants, especially preterm infants, for whom it is particularly beneficial [23]. Human milk can also help to achieve feeding milestones earlier compared to formula-fed infants [24]. This can be attributed to better feeding tolerance, immunoprotective and growth factors and maturation of immature host defenses [16,23].

Most preterm infants achieve oral feeding skills by 36–38 weeks postmenstrual age. However, extremely preterm infants (<28 weeks) and infants with medical complications need more time to attain this milestone [19,25]. Infants with younger gestational age have a significant delay in feeding initiation [19,24]. The most remarkable delay lies in the initiation, suggesting a maturational delay in neurological, gastrointestinal and/or suck-swallow-respiration coordination functions. Strikingly, when these younger preterm infants initiate oral feeding, they need the same time (3–5 weeks) to achieve full oral feeding [24]. Attaining early first and full enteral feeding seems to facilitate later feeding milestones [19]. In a recent study, Zhang quantified the impact of non-nutritive sucking and oral stimulation on feeding performance in a randomized controlled trial setting. The authors hereby quantified that non-nutritive sucking and oral stimulation reduced the median transition time (*i.e.*, introduction of oral to independent oral feeding) by about one week [26]. Focusing on the infant’s feeding readiness seems to be important to achieve full oral feeding earlier. In this cue-based feeding practice, assessment of early feeding skills is essential to select effective therapeutic feeding interventions [27]. In medical conditions, like neurological diseases (periventricular leukomalacia, Intraventricular bleeding Grades 3–4) or bronchopulmonary diseases, oral feeding development is commonly delayed [24]. This is particular the case in severe cases of bronchopulmonary disease and in former preterm neonates following surgically-treated patent ductus arteriosus.

## 3. The Fate of Fat: Pre-Exposure Fat Losses during Nasogastric Tube Feeding

The need to use gavage feeding of preferably human milk in the setting of neonatal intensive care results in the practice of milk storage, pasteurization and subsequent administration by an infusion system [5]. However, infusion systems used for enteral nutrition support in preterm neonates result in a significant reduction of lipids (up to 40%) delivered to the intestinal track [28,29,30,31]. These major losses of lipids further add to initially more limited losses due to pasteurization [32,33,34], freezing and thawing [34,35].

**Figure 1:**
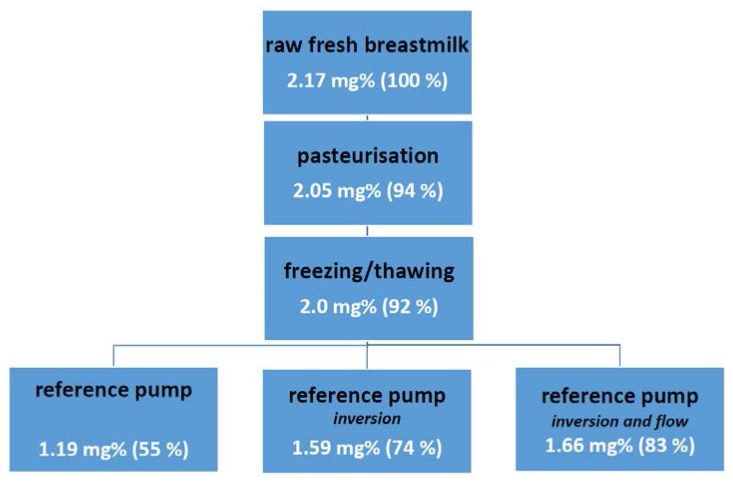
The progressive losses of fat content (mg%) in mother’s milk during the chain of processes commonly used in the NICU setting. The data were pooled from the publications of Vieira *et al.* on the impact of pasteurization and freezing/thawing and of Jarjour *et al.* on the impact of mixing of human milk (inversion, variable flow) during continuous infusion [29,34].

The extent of the impact and consecutive losses in lipids has been summarized in Figure 1. In addition to these quantitative effects, there are also some observations on qualitative effects, since adherence of medium-chain fatty acids to feeding tubes during gavage feeding for fortified human milk have also been described [36]. We were struck by the limited number of efforts made to prevent the major losses (80% of the pre-exposure lipid losses) related to the tube feeding techniques themselves, when compared to the observations on pasteurization or freezing/thawing (relative losses 20%), although the impact of pasteurization is not limited to lipid content only.

Avoidance of tube feeding by earlier introduction of oral feeding and preventive strategies related to switching from tube feeding to oral feeding have been discussed earlier. Avoidance of pasteurization in the setting of mother’s own milk seems a very reasonable option, when there is sufficient focus on collection, storage and labeling procedures to ensure the safety and quality of expressed milk [37]. Pasteurization does not only affect the lipid content, but also other characteristics, including bactericidal functions or functional elimination of lipase activity present in fresh human milk [38]. In preterm infants fed pasteurized human milk or formula, one week of treatment with recombinant human bile salt-stimulated lipase (rhBSSL) was well tolerated and significantly improved growth and long-chain polyunsaturated fatty acid absorption compared to placebo [38]. The mean weight increase was 17% higher (16.86 *vs.* 13.93 g/kg/day for one week) in the cases exposed. Unfortunately, the preliminary communication of the results of the subsequent phase III study (LAIF, lipase added to infant feeding, 410 infants, four weeks of exposure) failed to confirm these differences (16.8 *vs.* 16.6 g/kg/day, *p* = 0.49) [39]. 

**Figure 2:**
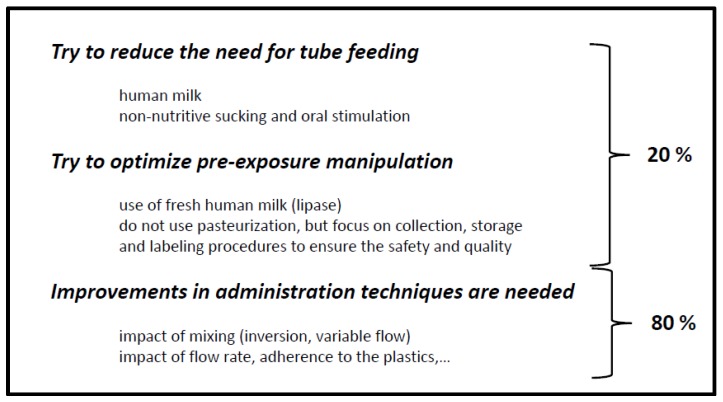
Strategies to reduce pre-exposure fat losses during nasogastric tube feeding. Relevant efforts have been made to improve the relative limited (20%) losses on tube feeding and pre-exposure manipulation, while there is still much to gain through improvements (80% of relative fat losses) in administration techniques.

In contrast, concepts to avoid major lipid losses due to tube feeding itself remained underexplored. At least the most recent Cochrane analysis on continuous *vs.* intermittent bolus milk feeding for premature infants (<1500 g) could not document any benefit (e.g., growth, necrotizing enterocolitis) [40]. However, a potential relevant technical concept has been reported in this journal [29]. To address the separation of fat from aqueous milk in the feeding bag, an *in vitro* motorized feeding bag inverter was developed. Three inversions of the milk-containing bag were applied during ten seconds, followed by a three-minute interval of rest before the next set of inversions. Additionally, the authors designed a circulation loop to circulate fast-flowing milk through the tubing back to the feeding bag in order to minimize fat separation and adherence in the ePumpTM infusion set tubing. Compared to the standard practices (60% of milk fat), the use of a feeding bag inverter and a tubing circulation loop delivered about 90% of milk fat when used in conjunction with a commercial continuous infusion system. This report was limited to *in vitro* observations, but warrants prospective evaluation, preferably by randomized controlled trials with a focus on relevant outcome variables (weight gain, neurocognitive outcome) [29]. Recently, Tabata *et al.* illustrated fat loss in human milk, the influence of added nutrients and the method of infusion [41]. Fat loss was greater when human milk was administered with a vertical feeding bag, compared to a horizontal syringe, providing a benefit for the horizontal syringe method of infusion. Whenever possible, the shortest time of infusion is preferred (e.g., gravity driven). For those infants who do not seem to tolerate their feeds well, Tabata’s study provides evidence that the addition of human milk fortifier (HMFand/or cream) increases the fat concentration of the human milk when given over a one-hour infusion. Potential strategies to reduce pre-exposure fat losses are summarized in Figure 2.

Another reason why intermittent bolus feeding should be preferred is the fact that it promotes physiological, cyclical surges of gastro-intestinal hormones [42]. Recently, it was shown that bolus feeding enhances protein synthesis and promotes greater protein deposition [43]. This is due to the increase of insulin and circulating amino acids, hereby activating the intracellular signaling pathways, which leads to mRNA translation.

## 4. Similarities between Developmental Drug and Developmental Nutritional Product Development

When writing this commentary, we were struck by the similarities in product development in the fields of developmental pharmacology and developmental nutritional support. Both aim to develop more population-tailored products and, therefore, need to integrate the maturational needs of the preterm neonate (e.g., maturational absorption, maturational caloric needs), as well as pre-administration manipulations that also may affect the real, final exposure to a given compound (product stability, compatibility, adherence to tubing). Drug administration in neonates presents a series of challenges that relate to the pathophysiology of the neonate (dose selection, developmental pharmacokinetics and dynamics) [44,45] and the systems or methods to administer these doses accurately. To illustrate this for intravenous formulations, several challenges arise, e.g., slow flow rates, small volumes, dead space volume and limitations on the total volume. While there is a reasonable understanding of neonatal pharmacokinetics [46], an appreciation of the substantial delay and variability in the rate of drug delivery from the intravenous line to the neonate is often lacking. Besides drug-related issues, like compatibility or density, delivery-related issues (e.g., dead space, fluid flow dynamics, inline filters or tubing diameter, components in the tubing, flow rate) for either drugs or nutritional compounds do have similar issues. We suggest that the available practices for “*in vitro*” drug evaluations should be considered to further improve the nutritional status and outcome of preterm neonates. For example, the potential impact of, e.g., simultaneous administration with milk or adherence to the nasogastric tube of drug formulations has also been recognized by the authorities involved in drug registration, since this is included in their guidelines [47].

## 5. Conclusions

Deficient nutritional support and subsequent postnatal growth failure are major covariates of short- and long-term outcome in preterm neonates. Despite its relevance, extrauterine growth restriction (EUGR) is still prevalent, occurring in an important portion of extremely preterm infants. Driven by (i) the need to switch as soon as possible from parenteral to enteral feeding, (ii) in the setting of preterm neonates who are not yet able to tolerate oral intake and with (iii) human milk that is suboptimal in preterm neonates, nasogastric tube feeding of fortified, manipulated human milk (freezing and thawing, pasteurization) is the standard approach until preterm neonates develop the capacities for full oral intake. Unfortunately, all of these pre-exposure practices significantly affect the final extent of lipid disposition in the intestinal track available for absorption, with the use of tube feeding being the most significant contributor.

Clearly, strategies to shift earlier to oral feeding (e.g., the impact of nonnutritive sucking and of oral stimulation on feeding performance in preterm infants, cue-based feeding) need to be a high priority., Studies on the impact of pasteurization are available, but may not be relevant in the setting of mother’s own milk when there is sufficient focus on collection, storage and labeling procedures to ensure the safety and quality of expressed milk. In contrast, and despite the fact that the use of tube feeding is the most significant contributor, studies on adaptations of infusion systems (inversion, variable flow) have only more recently been shown to be effective in “*in vitro*”, but not yet in “*in vivo*” settings. Since pre-exposure related issues for drugs or nutritional compounds show similarities, we suggest that the available practices for “*in vitro*” drug evaluations should be considered to further improve the nutritional status and outcome of preterm neonates. 
